# Levels and changes in cognitive, mental, and physical health as correlates of attitudes to aging in very old age

**DOI:** 10.3389/fpsyt.2025.1567754

**Published:** 2025-07-11

**Authors:** Serena Sabatini, Katya Numbers, Nicole A. Kochan, Perminder S. Sachdev, Henry Brodaty

**Affiliations:** ^1^ School of Psychology, Faculty of Health and Medical Sciences, University of Surrey, Guildford, United Kingdom; ^2^ Department of Clinical Psychology and Psychobiology, University of Barcelona, Barcelona, Spain; ^3^ Centre for Healthy Brain Ageing (CHeBA), Discipline of Psychiatry & Mental Health, School of Clinical Medicine, Faculty of Medicine & Health, University of New South Wales, Sydney, NSW, Australia; ^4^ Neuropsychiatric Institute, The Prince of Wales Hospital, Randwick, NSW, Australia

**Keywords:** subjective aging, self-perceptions of aging, mental health, physical health, cognition

## Abstract

**Introduction:**

Studies investigating the associations between change in health indicators and multidimensional measures of self-perceptions of aging in very old age are scarce. This study investigated whether levels of and 12-year changes in objective and subjective indicators of cognitive, mental, and physical health explain variance in the self-perceptions of aging of very old individuals at follow-up.

**Methods:**

Participants were 174 individuals enrolled in the Australian Memory and Aging Study (mean age = 87.41; SD = 3.67; 60% women). As health indicators, we used global cognition, the Memory Assessment Clinic Questionnaire, the Goldberg Anxiety Scale, the Geriatric Depression Scale, number of diagnosed health conditions, and self-rated health. Self-perceptions of aging were assessed with the Laidlaw Attitudes to Aging Questionnaire, which comprises three subscales capturing perceived psychological growth, psychosocial loss, and positive physical change. Simple and multivariable linear regression models were estimated.

**Results:**

Cross-sectionally, in multivariable linear regression models, more anxiety symptoms were associated with higher psychosocial loss (*R* ^2^ = 6%) and higher self-rated health (*R* ^2^ = 14%) was associated with higher perceived positive physical change. Cognition was not significantly associated with attitudes to aging subscales. Longitudinally, less increase in depressive symptoms was associated with less perceived psychosocial loss (*R* ^2^ = 5%) and with greater perceived positive physical change (*R* ^2^ = 11%).

**Conclusion:**

Self-perceptions of aging in different domains are cross-sectionally associated with different health indicators. However, among cognitive, mental, and physical health indicators, changes in depressive symptoms are the most correlated with perceived psychosocial loss in very old age.

## Introduction

Self-perceptions of aging (SPA) is an umbrella term that describes an individual’s subjective experiences, beliefs, and evaluations of their own aging and of the changes they experience in their lives as they age (1). Individuals can have both positive and negative SPA. Examples of positive SPA are recognizing increased knowledge and life experience, more confidence, and greater ability to deal with people, while examples of negative SPA are experiencing poorer memory, greater dependency on others, and less energy (2). Although it is natural for negative SPA to increase with biological aging, positive SPA can still be experienced even in later years (3). Moreover, levels of negative SPA can vary in older age (4). Generally, having more positive SPA and fewer negative SPA is considered beneficial in later life (5). This is because positive SPA can shape individuals’ behavior (adopting a healthier lifestyle), physical health (via immunocompetence), and emotional wellbeing (thinking and feeling better) (6). Therefore, promoting positive SPA and decreasing negative SPA may help promote active and healthy aging (7, 8).

Positive SPA show small to moderate associations with subjective and objective indicators of health over time, with associations being stronger with subjective indicators (e.g., self-rated health and perceived cognition) compared to objective indicators (9, 10). Moreover, positive SPA are consistently associated with lower risk of mental and physical health conditions and lower risk of disability, including lower risk of frailty, falls, and functional difficulties (5, 11–13). More positive SPA are also associated with better biological aging (14). These results are supported by a comprehensive meta-analysis comprising more than 100 longitudinal studies with a median observational interval of approximately 5 years (9). The effect of SPA on longevity has been compared to the effect of established risk factors such as smoking or obesity (15). Furthermore, more positive SPA are associated with better objectively assessed and informant-rated cognitive functioning and lower dementia risk (5, 13).

SPA may impact health through psychological, behavioral, and physiological pathways (16). The psychological pathway describes how more positive SPA may increase self-efficacy, adaptive self-regulation, will to live, and an open and positive outlook toward the future (17, 18). The behavioral pathway describes how SPA influences engagement in health-enhancing behaviors, adaptive behaviors, and social behaviors (19, 20). For example, positive SPA are associated with better outcomes such as greater future engagement with social and leisure (e.g., sport, reading, and charity work) activities (21, 22). Lastly, the physiological pathway describes how particularly negative SPA are associated with negative biological processes like increased inflammation, which can lead to worse health-related conditions (14).

Given the prevalence of negative SPA (e.g., 56% in a sample of 83,034 participants from 57 countries), the impact of SPA on peoples’ lives, the healthcare system, and economy could be substantial (23). For example, Levy and colleagues estimated that in the US, negative views on aging and SPA taken together are the cause of 17.0 million cases of newly diagnosed health conditions each year, resulting in an annual cost of $63 billion (24). Identifying predictors of SPA is important as tackling SPA and its predictors can help promote healthy aging. A limited but increasing amount of research suggests that the associations between SPA and health indicators are bidirectional. That is, the positive and negative changes that individuals may experience in their health and lifestyle/daily life can also influence their SPA. Increases in symptoms of depression and anxiety, newly diagnosed health conditions (e.g., cancer, cardiovascular events and hypertension), and greater functional difficulties may all lead to more negative SPA (25–28).

Most longitudinal evidence focuses on the unidimensional measures and not on the multidimensional measures of SPA (9). Indeed, there are both unidimensional (e.g., Felt Age and Attitudes Towards Own Aging) and multidimensional (e.g., Aging-Related Cognitions Scales, Awareness of Age-Related Change, and Attitudes to Aging) concepts and measures of SPA (29, 30). Compared to unidimensional measures, multidimensional measures have the advantage that they allow for a separate assessment of both positive and negative SPA. This is important, as in each phase of the lifespan, individuals experience both gains and losses, though in very old age, the objective and subjective experience of gains typically decreases and losses increase (3, 27).

Moreover, some multidimensional measures of SPA [such as the Awareness of Age-Related Change questionnaire (31) and the Attitudes to Aging Questionnaire (AAQ)] make it possible to obtain separate scores for the age-related changes individuals experience in different domains of their lives. In doing so, they make it possible to investigate whether perceived age-related changes in one domain are associated with objective changes in that same domain. One of the more widely used multidimensional measures of SPA is the AAQ, which exists in a long (24 items) and short (12 items) form (32–34). The questionnaire assesses three domains associated with aging attitudes: psychosocial loss (sample item: *As I get older, I find it more difficult to make new friends; answer option: 1 = strongly disagree; 5 = strongly agree*), positive physical health change (sample item: *My health is better than I expected for my age; answer option: 1 = strongly disagree; 5 = strongly agree*), and psychological growth (sample item: *It is a privilege to grow old; answer option: 1 = strongly disagree; 5 = strongly agree*). The AAQ (34) was specifically developed for people aged over 60 as it contains items that directly question respondents’ experiences of aging such as “I am losing my independence as I get older”.

To the best of our knowledge, there are only a few longitudinal studies that have investigated outcomes associated with aging attitudes using the AAQ, and their findings have been mixed. One study using data from the English Longitudinal Study of Aging found that older people with more positive attitudes to aging (with regard to physical health change and psychological loss) have reduced risk of becoming physically frail or pre-frail (35). Another study using data from the UK Lothian Birth Cohort 1936 found that none of the AAQ subscales were predictive of sedentary or walking behaviors 7 years later (36). A final study, also using data from the UK Lothian Birth Cohort, found that only more positive scores on one subscale of the AAQ—attitudes towards physical change—were associated with lower mortality (37). This is not surprising as the psychosocial loss and psychological growth subscales do not assess aspects directly related to health and mortality.

Fewer studies have explored what are the correlates of SPA using the AAQ. One study, again using data from the UK Lothian Birth Cohort, found that predictors of worse SPA in the psychosocial loss domain were personality (e.g., high neuroticism and low extraversion), mood (e.g., anxiety and depression), and more physical disability (38). In the same study, predictors of more positive SPA in the physical change domain were high extraversion, openness, agreeableness, conscientiousness, female sex, higher social class, and less physical disability. Predictors associated with more positive SPA in the psychological growth domain were similar to those associated with positive physical change, whereas being less affluent, living alone, worse vocabulary, and slower walking speed predicted more negative SPA in the psychological growth domain. However, because the AAQ and health correlates were assessed at different waves, the authors did not investigate whether changes in mental, physical, and cognitive health over time were associated with AAQ at follow-up.

The aims of the current study are, first, to investigate in a sample of older Australians cross-sectional associations between a range of objective and subjective indicators of cognitive, mental, and physical health (i.e., global cognition, memory complaints, anxiety symptoms, depressive symptoms, number of health conditions, and self-rated health) and scores on the AAQ’s three subscales (i.e., psychological growth, psychosocial loss, and positive physical change). Next, the current study aims to determine whether 12-year changes in these same objective and subjective indicators of cognitive, mental, and physical are associated with follow-up scores on the AAQ’s subscales.

We hypothesize that:

1. Higher scores on the psychological growth and positive physical change subscale and lower scores on the psychosocial loss subscale of the AAQ will be associated with better cognitive, mental, and physical health indicators, cross-sectionally. This would be in line with existing evidence linking more positive and less negative SPA to better cognitive, mental, and physical health (5, 9, 10).
2. Less 12-year decline in cognitive, mental, and physical health indicators is associated with higher follow-up psychological growth, more positive follow-up physical change, and lower follow-up psychosocial loss. Again, this would be in line with the limited evidence available showing that decline in cognitive, mental, and physical health can act as antecedents of SPA (11, 25).
3. Self-reported health indicators will show stronger associations with psychological growth, psychosocial losses, and positive physical changes than objective health indicators, as both self-rated health and AAQ’s subscales are subjective variables. This is also based on previous cross-sectional and longitudinal evidence reporting stronger associations between SPA and subjective indicators of health compared to objective indicators of health (25, 39).
4. Although existing evidence linking specific health indicators with measures/scales assessing SPA in the same domain is scarce (39–41), we expect stronger associations between specific health indicators and the corresponding SPA subscale domains (e.g., depression and the psychological growth subscale) than between unrelated health indicators and AAQ’s subscales. Indeed, the possible utility of domain-specific measures of SPA in predicting matched outcomes has previously been reported by Levy and Leifheit-Limson (41).

## Materials and methods

Participants were from the Sydney Memory and Aging Study (MAS) (42), one of Australia’s largest studies of aging and cognitive health. MAS participants were recruited via the Australian electoral roll from two local government areas in Sydney’s Eastern suburbs from 2005 to 2007. As Australia has compulsory voting, electoral rolls comprise nearly all citizens. Baseline exclusion criteria were (a) insufficient fluency in English to complete a psychometric assessment; (b) a Mini-Mental State Examination (MMSE) (43) score < 24 after adjusting for age, education, and non-English speaking background; and (c) diagnoses of a major neurological illness such as dementia, motor neuron disease, or progressive malignancy. More details regarding baseline characteristics have been published previously (42). Participants were assessed biennially over 12 years, with each assessment called a “wave”. At each wave, participants completed questionnaires about their health, wellbeing, and mood, underwent a comprehensive neuropsychological assessment, and had a brief medical assessment. The AAQ was only added to the MAS protocol at wave 7 (12-year follow-up in 2021). Of the initial MAS sample of 1,053 participants, only 309 (29.8%) were included in wave 7, whereas 405 (39.1%) were deceased, 26 (2.5%) were lost to follow-up, 252 (24.3%) had withdrawn, only informant data were available for 45 (4.3%), and data were missing for 16 participants. Compared to MAS participants who took part at wave 7, at baseline, those who did not take part at wave 7 (due to withdrawal, having died, or having been lost to follow-up) were significantly older, included a significantly higher proportion of men, had slightly better global cognition, had almost one more depressive symptom each, and rated their health slightly more poorly (**Supplementary Table S1**). The current study sample includes only individuals who participated in wave 7 of MAS and who also completed the AAQ. Hence, we included 174 participants in the current analyses. Importantly, the AAQ questionnaire was not administered to all participants of MAS wave 7 due to the insurgence of COVID-19 and the consequent interruption of data collection. Compared to participants who completed the AAQ at wave 7, those who did not complete the AAQ at wave 7 were significantly older, included fewer individuals who were married and more who were widowed, and had poorer global cognition (**Supplementary Table S2**). Compared to those who completed the AAQ at wave 7, those who did not complete the AAQ at wave 7 had better global cognition at wave 7, but had slightly poorer self-rated health (**Supplementary Table S3**). In the current study, cross-sectional analyses used wave 7 measures. Longitudinal changes in cognitive, mental, and physical health indicators were retrospectively calculated over the previous 12 years. This study was approved by the University of New South Wales Human Research Ethics Committee (HC: 200671, 05037, 09382, 14327, and 90626) in accordance with the National Statement on Ethical Conduct in Human Research (2007) and the Declaration of Helsinki. Informed written consent was obtained from all participants prior to participation.

### Measures

#### Aging attitudes

The 12-item AAQ (34) was used to assess participants’ SPA. The AAQ is a validated instrument comprising three subscales assessing perceived psychological growth, psychosocial loss, and positive physical change. Each subscale contains four items, rated on a five-point Likert scale ranging from 1 (strongly disagree) to 5 (strongly agree). The psychological growth subscale asks participants about positive things they may be experiencing while aging, such as continued development and satisfaction. The psychosocial loss subscale assesses negative SPA, including feelings of social loss and declining self-worth. The positive physical change subscale evaluates attitudes towards one’s physical changes associated with aging, including health and fitness. Scores for each subscale are calculated by summing the responses to the four items, resulting in a range of 4 to 20 for each subscale. Higher scores on the psychological growth and physical change subscales indicate more positive SPA, whereas higher scores on the psychosocial loss subscale reflect more negative SPA. In the current study sample, Cronbach’s alpha for the perceived physical change scale was 0.70, that for the perceived psychosocial loss scale was 0.74, and that for the perceived psychological growth scale was 0.75.

#### Cognitive health indicators

##### Global cognition

Global cognition composite scores were derived from participants’ scores on a comprehensive neuropsychological test battery consisting of 10 tests measuring attention, language, executive function, visuospatial ability, memory, and verbal memory domains. Domain and global cognition composites were presented as standardized *z*-scores. Raw test scores were converted to *z*-scores using the means and standard deviations (SDs) from a reference group of 723 MAS participants classified as cognitively healthy at baseline. This reference group included native English speakers with MMSE (43) scores of 24 or higher, no evidence of dementia or current depression, no history of delusions or hallucinations, and no major neurological diseases or significant head injuries. Composite domain scores were calculated by averaging the *z*-scores of the component tests. Global cognition scores at baseline and at 6 years’ follow-up were derived by averaging the domain scores. All domain and global cognition scores were standardized to the mean and SD (0 and 1, respectively) of the baseline reference group. Additional details on the calculation of cognitive domain and global cognition scores, and the specific tests comprising each cognitive domain, are provided in **Supplementary Text 1** and **Supplementary Table S4**.

##### Subjective memory complaints

Subjective memory complaints were captured using the Memory Assessment Clinic Questionnaire (MAC-Q) (44). The questionnaire comprises six items, and each item is scored on a five-point Likert scale (1 = much better now; 2 = somewhat better now; 3 = about the same; 4 = somewhat poorer now; 5 = much poorer now). Items ask participants whether they have difficulties remembering the name of a person just introduced to them; recalling telephone numbers or post codes that they use on a daily or weekly basis; recalling where they have put objects (such as keys) in their home or office; remembering specific facts from a newspaper or magazine article they have just finished reading; remembering the items they intended to buy when they arrive at the supermarket; and how they would describe their memory as compared to 10 years ago in general. In the current study sample, Cronbach’s alpha was 0.95 for the MAC-Q.

#### Mental health indicators

##### Mood

The Goldberg Anxiety Scale (GAS) (45) was used to assess anxiety symptoms. It comprises nine items. Example items are “have you felt keyed up or on edge” and “have you been worrying a lot.” For each item, participants can answer either yes or no. The total score consists of the sum of items/yes. In the current study sample, Cronbach’s alpha for the GAS was 0.71. The Geriatric Depression Scale (GDS) (46) was used to assess depressive symptoms over the past week. It comprises 15 items. Sample items are “are you basically satisfied with your life?” and “do you feel your situation is hopeless?”. For each item, participants can answer either yes or no. The total score consists of the sum of items/yes. In the current study sample, Cronbach’s alpha for the GDS was 0.98.

#### Physical health indicators

##### Medical comorbidities

The number of participants’ health conditions was counted based on reports of 17 health conditions between wave 1 and wave 7 of the MAS study. Examples of included conditions are stroke/cerebrovascular accident, diabetes, and respiratory or lung disease. The total possible score ranges from 0 to 17.

##### Self-rated health

Self-rated health was assessed with a single-item question: “In general, would you say your health is…?” Participants could choose from five answer options: 1 = poor, 2 = fair, 3 = good, 4 = very good, and 5 = excellent.

### Covariates

Socio-demographic variables comprised age, sex (women and men), and marital status (never married, married de facto, separated, divorced, and widowed) at follow-up. Main occupation when working/before retirement (manager admin, professional, associate professional, tradesperson, advanced clerical service, intermediate clerical sales service, intermediate production and transport, elementary clerical/sales service, intermediate laborers and related, and home duties) was also assessed. Race (Caucasian and other) was reported solely to describe the study samples.

### Analyses

Correlations among study variables were calculated using Spearman’s correlation coefficients for categorical variables and Pearson’s correlation coefficients for continuous variables.

We conducted linear regression models to investigate whether each of the study variables (i.e., global cognition, memory complaints, anxiety symptoms, depressive symptoms, number of health conditions, and self-rated health) was cross-sectionally associated with each of the AAQ subscales psychological growth, psychosocial losses, and positive physical change. For each predictor, we conducted an unadjusted model (model 1) and a model adjusted for age, sex, marital status, and main occupation when working (model 2). We also conducted a third model comprising, in addition to covariates, all investigated cross-sectional predictors.

To investigate whether 12-year changes in cognitive, mental, and physical health between wave 1 and wave 7 explain variance in attitudes to aging at wave 7 (follow-up), we first calculated a change score for each health indicator. To calculate change scores, we subtracted the value at follow-up from the value at baseline. We then used linear regression models to investigate whether 12-year change scores in each health indicator were associated with values on each of the AAQ subscales psychological growth, psychosocial losses, and physical change at wave 7 while adjusting for baseline cognitive scores (i.e., participants’ scores on global cognition). Similarly to cross-sectional analyses, for longitudinal analyses, we estimated an unadjusted model (model 1); a model adjusted for age, sex, marital status, main occupation when working, and baseline levels of cognition (model 2); and a model comprising, in addition to covariates, all investigated predictive variables. The choice of using change scores, coupled with baseline adjustment and covariates, offers a parsimonious and theoretically valid approach, minimizing assumptions about the shape of change while directly addressing the research question.

To quantify the associations, we reported standardized regression coefficients (β, effects sizes), in addition to unstandardized regression coefficients. Values ≤ 0.09 were considered very small; 0.10–0.29, small; 0.30–0.49, moderate; and ≥0.50, large (47). Complete case analyses were conducted. We conducted all analyses in STATA version 17.

## Results

### Descriptive statistics at wave 7

There were 174 participants. Their mean (M) age at wave 7 was 87.41 (SD = 3.67); 59.77% were women (**Table 1**). At baseline, participants were aged between 70 and 90 years, and at the 12-year follow-up (wave 7), participants for the current study were aged between 83 and 97 years. All participants reported that they were retired. Before retirement, 13.9% worked as a manager, 38.7% worked as a professional, 6.9% worked as an associate professional, 2.9% worked as a tradesperson, 11.6% worked in the advanced clerical service sector, 16.8% worked in the intermediate clerical sales service field, 0.6% worked in the intermediate production and transport sector, 4.6% worked in the elementary clerical/sales service field, 0.6% worked as laborers and related professions, and 3.4% did home duties. Most participants were Caucasian (99.4%). Participants’ mean score on global cognition was −0.49 (SD = 0.82). Participants’ mean score on MAC-Q was 24.06 (SD = 3.35), indicating the presence of several memory complaints. On average, participants reported few anxiety (M = 2.31, SD = 2.13) and depressive (M = 2.87, SD = 2.37) symptoms. On average, participants had between two and three health conditions and most perceived their health as good or very good.

**Table 1:** Descriptive statistics for the study sample.

Variables	Wave 7	Change score (wave 1 − wave 7)
*n*	174	
Age, M (SD)	87.41 (3.67)	
Sex, *n* (%)		
Women	104 (59.77)	
Men	70 (40.23)	
Marital status, *n* (%)		
Never married	16 (9.1)	
Married de facto	72 (41.3)	
Separated	1 (0.5)	
Divorced	17 (10.0)	
Widowed	68 (39.1)	
Main occupation when working, *n* (%)		
Manager admin	24 (13.9)	
Professional	67 (38.7)	
Associate professional	12 (6.9)	
Tradesperson	5 (2.9)	
Advanced clerical service	20 (11.6)	
Intermediate clerical sales service	29 (16.8)	
Intermediate prod transport	1 (0.6)	
Elementary clerical, sales service	8 (4.6)	
Laborers and related	1 (0.6)	
Home duties	6 (3.4)	
Missing	1	
Race, *n* (%)		
Caucasian	172 (99.4)	
Other	1 (0.6)	
Missing	1	
Global cognition, M (SD)	−0.49 (0.82)	0.97 (0.66)
Missing	71	
Memory complaints, M (SD)	24.06 (3.35)	−0.37 (3.33)
Missing	10	
Anxiety symptoms, M (SD)	2.31 (2.13)	−1.42 (2.21)
Missing	14	
Depressive symptoms, M (SD)	2.87 (2.37)	−1.34 (2.17)
Missing	16	
Number of health conditions, M (SD)	2.6 (1.6)	−1.48 (1.64)
Self-rated health, M (SD)	3.19 (0.91)	0.43 (1.04)
Poor, n (%)	4 (2.4)	
Fair, n (%)	36 (21.7)	
Good, n (%)	57 (34.3)	
Very good, n (%)	62 (37.4)	
Excellent, n (%)	7 (4.2)	
Missing	8	

Global cognition declined over 12 years. The mean change score for global cognition was 0.97 (SD = 0.66). Perceived memory issues increased over 12 years, and the mean change score for memory issues was −0.37 (SD = 3.33). Over the 12-year study period, anxiety (M change = −1.42, SD = 2.22) and depressive (M change = −1.34, SD = 2.17) symptoms increased. The number of health conditions on participants increased, on average, by 1.48 over 12 years, and self-rated health become poorer.

### Correlations among study variables at wave 7

Correlations among study variables are reported in **Supplementary Table S5**. Starting from outcome variables, perceived psychological growth was associated with more perceived positive physical change, fewer memory complaints, and fewer depressive symptoms. All correlations were of small size. More perceived psychosocial loss was correlated with lower perceived positive physical change, being men, more anxiety symptoms, and poorer self-rated health. All correlations were of small size. Higher perceived positive physical change was correlated to younger age, being men, better global cognition, fewer anxiety symptoms, and better self-rated health. All correlations were of small size except from the correlation with self-rated health, which was of moderate size. Among predictive variables, global cognition was moderately correlated with better self-rated health. Memory complaints were correlated to a small extent with more depressive symptoms and poorer self-rated health. More anxiety and more depressive symptoms were correlated to a moderate extent with poorer self-rated health. A greater number of health conditions were correlated to a small extent with poorer self-rated health.

### Cross-sectional analyses

#### Associations between health indicators and psychological growth

In adjusted linear regression models, global cognition, anxiety symptoms, number of health conditions, and self-rated health were not significantly associated with perceived psychological growth (**Table 2**; Model 2). More memory complaints were significantly associated with poorer perceived psychological growth (Unstandardized Beta, *B* = −0.004, 95% CI: −0.01, −0.002, *p*-value = 0.002, *R* ^2^ = 6%, Standardized Beta, β= −0.24). More depressive symptoms were significantly associated with lower perceived psychological growth (*B* = −0.28, 95% CI: −0.55, −0.02, *p*-value = 0.036, *R* ^2^ = 0.2%, β = −0.17). Effects were of small size. When including all correlates in a multidimensional model, none of the investigated variables were significantly associated with psychological growth.

**Table 2:** Cross-sectional associations between indicators of cognitive, mental, and physical health and psychological growth.

Psychological growth as outcome									
Variables	Model 1. Unadjusted linear regression			Model 2. Adjusted linear regression			Model 3. Multivariable linear regression including all predictors		
	*B* (95% CI), *p*-value	β	*R* ^2^	*B* (95% CI), *p*-value	β	*R* ^2^	*B* (95% CI), *p*-value	β	*R* ^2^
Global cognition	0.61 (−0.35, 1.56), 0.209	0.12	0.02	0.70 (−0.28, 1.67), 0.158	0.14	0.02	0.61 (−0.49; 1.70), 0.275	0.12	0.01
Memory complaints	−0.004 (−0.01, −0.001), 0.003	−0.23	0.05	−0.004 (−0.01, −0.002), 0.002	−0.24	0.06	0.10 (−0.17; 0.36), 0.461	0.08	0.01
Anxiety symptoms	0.02 (−0.28, 0.32), 0.903	0.01	0.001	0.03 (−0.28, 0.33), 0.872	0.01	0.002	0.21 (−0.25; 0.66), 0.366	0.10	0.01
Depressive symptoms	−0.28 (−0.54, −0.02), 0.036	−0.17	0.03	−0.28 (−0.55, −0.02), 0.036	−0.17	0.03	0.001 (−0.01; 0.01), 0.764	0.03	0.001
Number of health conditions	0.30 (−0.15, 0.74), 0.190	0.10	0.01	0.31 (−0.14, 0.77), 0.178	0.10	0.01	0.26 (−0.29; 0.81), 0.354	0.10	0.01
Self-rated health	0.49 (−0.19, 1.17), 0.156	0.11	0.01	0.47 (−0.22, 1.16), 0.177	0.11	0.01	0.64 (−0.40; 1.68), 0.224	0.14	0.02

Models 2 and 3 are adjusted for age, sex, marital status, and occupation before retirement. *N* = 174. *B* = Unstandardized regression coefficient. β = Standardized regression coefficient.

#### Associations between health indicators and psychosocial loss

In adjusted linear regression models, global cognition, memory complaints, depressive symptoms, and number of health conditions were not significantly associated with perceived psychosocial loss (**Table 3**; Model 2). More anxiety symptoms were associated with higher perceived psychosocial loss (*B* = 0.65, 95% CI: 0.36, 0.95, *p*-value < 0.001, *R* ^2^ = 34, β = 0.11). Importantly, anxiety symptoms explained 34%—a substantial amount—of the variance in psychosocial loss. Poorer self-rated health was associated with greater perceived psychosocial loss (*B* = −0.97, 95% CI: −1.64, −0.29, *p*-value = 0.005, *R* ^2^ = 5%, β = −0.22). When including all correlates in a multidimensional model, only more anxiety symptoms remained significantly associated with perceived psychosocial loss (*B* = 0.57, 95% CI: 0.14, 1.00, *p*-value = 0.010, *R* ^2^ = 6%, β = 0.28) and the variance they explained in psychosocial loss decreased from 34% to 6%.

**Table 3:** Cross-sectional associations between indicators of cognitive, mental, and physical health and psychosocial loss.

Psychosocial loss as outcome									
Variables	Model 1. Unadjusted linear regression			Model 2. Adjusted linear regression			Model 3. Multivariable linear regression including all predictors		
	*B* (95% CI), *p*-value	β	*R^2^*	*B* (95% CI), *p*-value	β	*R^2^*	*B* (95% CI), *p*-value	β	*R^2^*
Global cognition	−0.47 (−1.40, 0.46), 0.316	−0.10	0.01	−0.21 (−1.16, 0.74), 0.664	−0.04	0.02	−0.39 (−1.38; 0.61), 0.441	−0.08	0.01
Memory complaints	0.002 (−0.001, 0.004), 0.186	0.10	0.01	0.001 (−0.002, 0.004), 0.452	0.06	0.003	0.18 (−0.06; 0.42), 0.142	0.15	0.02
Anxiety symptoms	0.54 (0.24, 0.83), <.001	0.28	0.80	0.65 (0.36, 0.95), <.001	0.11	0.34	0.57 (0.14; 1.00), 0.010	0.28	0.06
Depressive symptoms	−0.001 (−0.003, 0.002), 0.643	−0.04	0.001	−0.001 (−0.003, 0.002), 0.621	−0.04	0.001	−0.01 (−0.01; 0.002), 0.197	−0.13	0.02
Number of health conditions	0.30 (−0.09, 0.69), 0.128	0.12	0.01	0.25 (−0.14, 0.65), 0.203	0.10	0.01	0.24 (−0.26; 0.74), 0.336	0.10	0.01
Self-rated health	−0.90 (−1.58, −0.22), 0.010	−0.20	0.04	−0.97 (−1.64, −0.29), 0.005	−0.22	0.05	0.13 (−0.80; 1.06), 0.787	0.03	0.001

Models 2 and 3 are adjusted for age, sex, marital status, and occupation before retirement. *N* = 174. *B* = Unstandardized regression coefficient. β = Standardized regression coefficient.

#### Associations between health indicators and physical change

In adjusted linear regression models, higher scores on global cognition were associated with higher scores on perceived positive physical change (*B* = 0.81, 95% CI: 0.02, 1.61, *p*-value = 0.046, *R* ^2^ = 4%, β = 0.20, **Table 4**; Model 2). More anxiety symptoms were associated with lower perceived positive physical change (*B* = −0.40, 95% CI: −0.66, −0.14, *p*-value = 0.003, *R* ^2^ = 5%, β = −0.24). Better self-rated health was significantly associated with higher perceived positive physical change (*B* = 1.70, 95% CI: 1.18, 2.23, *p* < 0.001, *R* ^2^ = 19%, β = 0.44), and it explained a relevant amount of variance in perceived physical change (19%). Effects were of moderate size for self-rated health and of small size for the remaining significant predictors. Memory complaints, depressive symptoms, and the number of health conditions were not significantly associated with perceived positive physical change. In the multidimensional model including all correlates of physical change, only more positive self-rated health was significantly associated with higher scores on perceived positive physical change (*B* = 1.59, 95% CI: 0.80, 2.38, *p*-value = 0.001, *R* ^2^ = 14%, β = 0.42).

**Table 4:** Cross-sectional associations between indicators of cognitive, mental, and physical health and physical change.

Physical change as outcome									
Variables	Model 1. Unadjusted linear regression			Model 2. Adjusted linear regression			Model 3. Multivariable linear regression including all predictors		
	*B* (95% CI), *p*-value	β	*R* ^2^	*B* (95% CI), *p*-value	β	*R* ^2^	*B* (95% CI), *p*-value	β	*R* ^2^
Global cognition	0.99 (0.22, 1.78), 0.012	0.25	0.05	0.81 (0.02, 1.61), 0.046	0.20	0.04	0.28 (−0.56; 1.12), 0.511	0.07	0.004
Memory complaints	−0.001 (−0.003, 0.001), 0.411	−0.06	0.004	−0.001 (−0.004, 0.001), 0.225	−0.09	0.01	0.15 (−0.06; 0.35), 0.154	0.14	0.02
Anxiety symptoms	−0.44 (−0.70, −0.18), 0.001	−0.26	0.07	−0.40 (−0.66, −0.14), 0.003	−0.24	0.05	−0.20 (−0.57; 0.16), 0.268	−0.11	0.01
Depressive symptoms	−0.001 (−0.003, 0.001), 0.408	−0.06	0.004	−0.01 (−0.003, 0.002), 0.545	−0.05	0.002	0.003 (−0.003; 0.01), 0.362	0.09	0.01
Number of health conditions	−0.13 (−0.47, 0.21), 0.449	−0.06	0.003	−0.12 (−0.46, 0.22), 0.475	−0.05	0.003	0.07 (−0.35; 0.49), 0.730	0.03	0.001
Self-rated health	1.76 (1.24, 2.30), <0.001	0.46	0.21	1.70 (1.18, 2.23), <0.001	0.44	0.19	1.59 (0.80; 2.38), 0.001	0.42	0.14

Models 2 and 3 are adjusted for age, sex, marital status, and occupation before retirement. *N* = 174. *B* = Unstandardized regression coefficient. β = Standardized regression coefficient.

### Longitudinal analyses

#### Associations between 12-year change in health indicators and psychological growth

Change score in none of the investigated variables was significantly associated with perceived psychological growth in none of the investigated models (unadjusted, adjusted, and multivariable models; **Table 5**).

**Table 5:** Longitudinal associations between change in indicators of cognitive, mental, and physical health and psychological growth.

Change score	Model 1. Unadjusted linear regression			Model 2. Adjusted linear regression			Model 3. Multivariable linear regression including all predictors		
	*B* (95% CI), *p*-value	β	*R* ^2^	*B* (95% CI), *p*-value	β	*R* ^2^	*B* (95% CI), *p*-value	β	*R* ^2^
Global cognition	−0.60 (−1.80, 0.61), 0.328	−0.10	0.01	−0.69 (−1.87, 0.48), 0.244	−0.11	0.02	−0.77 (−2.09, 0.56), 0.254	−0.13	0.01
Memory complaints	−0.06 (−0.24, 0.13), 0.543	−0.05	0.002	−0.05 (−0.24, 0.14), 0.592	−0.04	0.003	−0.03 (−0.29, 0.24), 0.845	−0.02	0.0004
Anxiety symptoms	−0.08 (−0.37, 0.21), 0.601	−0.04	0.002	−0.09 (−0.39, 0.21), 0.569	−0.05	0.002	−0.11 (−0.54, 0.32), 0.620	−0.06	0.003
Depressive symptoms	0.23 (−0.06, 0.51), 0.117	0.13	0.02	0.24 (−0.06, 0.53), 0.111	0.13	0.02	−0.04 (−0.54, 0.45), 0.866	−0.02	0.0003
Number of health conditions	−0.01 (−0.39, 0.37), 0.947	−0.01	0.00	−0.04 (−0.43, 0.35), 0.847	−0.02	0.00	−0.08 (−0.64, 0.49), 0.793	−0.03	0.001
Self-rated health	0.15 (−0.46, 0.76), 0.629	0.04	0.001	0.15 (−0.45, 0.76), 0.618	0.04	0.002	−0.67 (−1.51, 0.17), 0.117	−0.67	0.03

Models 2 and 3 are adjusted for age, sex, marital status, and occupation before retirement. *N* = 174. *B* = Unstandardized regression coefficient. β = Standardized regression coefficient.

#### Associations between 12-year change in health indicators and psychosocial loss

In adjusted linear regression models, change score in global cognition, memory complaints, and number of health conditions were not significantly associated with perceived psychosocial loss (**Table 6**; Model 2). However, change score in anxiety symptoms was not significantly associated with perceived psychosocial loss. Greater increase in anxiety symptoms (*B* = −0.37, 95% CI: −0.67, −0.08, *p*-value = 0.013, *R* ^2^ = 4%, β = −0.20) and in depressive symptoms (*B* = −0.40, 95% CI: −0.69, −0.11, *p*-value = 0.008, *R* ^2^ = 4%, β = −0.21) was associated with higher perceived psychosocial loss. Greater decline in self-rated health was also associated with greater perceived psychosocial loss (*B* = −1.01, 95% CI: −1.50, 0.52, *p* = 0.001, *R* ^2^ = 9%, β = −0.30). Effects were of small size. In the multivariable regression model including all correlates of perceived psychosocial loss and covariates, only greater increase in depressive symptoms was significantly associated with greater perceived psychosocial loss (*B* = −0.52, 95% CI: −0.98, −0.06, *p*-value = 0.027, *R* ^2^ = 5%, β = −0.07).

**Table 6:** Longitudinal associations between change in indicators of cognitive, mental, and physical health and psychosocial loss.

Change score	Model 1. Unadjusted linear regression			Model 2. Adjusted linear regression			Model 3. Multivariable linear regression including all predictors		
	*B* (95% CI), *p*-value	β	*R* ^2^	*B* (95% CI), *p*-value	β	*R* ^2^	*B* (95% CI), *p*-value	β	*R* ^2^
Global cognition	0.61 (−0.55, 1.77), 0.301	0.10	0.02	0.44 (−0.72, 1.61), 0.453	0.08	0.01	−0.24 (−1.46; −0.99), 0.700	−0.04	0.002
Memory complaints	0.02 (−.0.16, 0.21), 0.797	0.02	0.0004	0.02 (−0.17, 0.22), 0.797	0.02	0.001	0.06 (−0.17; 0.30), 0.593	0.06	0.003
Anxiety symptoms	−0.26 (−0.56, 0.03), 0.080	−0.14	0.02	−0.37 (−0.67, −0.08), 0.013	−0.20	0.04	−0.38 (−0.77; 0.01), 0.057	−0.21	0.04
Depressive symptoms	−0.39 (−0.68, −0.09), 0.010	−0.21	0.04	−0.40 (−0.69, −0.11), 0.008	−0.21	0.04	−0.52 (−0.98; −0.06), 0.027	−0.27	0.05
Number of health conditions	−0.06 (−0.44, 0.32), 0.752	−0.02	0.001	−0.13 (−0.51, 0.24), 0.490	−0.05	0.003	−0.17 (−0.69; 0.35), 0.529	−0.15	0.004
Self-rated health	−0.98 (−1.47, −0.48), 0.001	−0.29	0.08	−1.01 (−1.50, 0.52), 0.001	−0.30	0.09	−0.50 (−1.24; 0.24), 0.182	−0.07	0.02

Models 2 and 3 are adjusted for age, sex, marital status, and occupation before retirement. *N* = 174. *B* = Unstandardized regression coefficient. β = Standardized regression coefficient.

#### Associations between 12-year change in health indicators and physical change

In the adjusted linear regression model, less decline in global cognition was significantly associated with higher perceived positive physical change (*B* = −1.06, 95% CI: −2.04, −0.08, *p* = 0.035, *R* ^2^ = 5%, β = −0.21, **Table 7**). Less increase in anxiety symptoms was associated with higher perceived positive physical change (*B* = 0.36, 95% CI: 0.11, 0.62, *p* = 0.006, *R* ^2^ = 5%, β = 0.22). Greater increase in self-rated health was significantly associated with higher perceived positive physical change (*B* = −1.01, 95% CI: −1.50, −0.52, *p* < 0.001, *R* ^2^ = 9%, β = −0.30). Effects were of small to moderate size. In the multivariable regression model including all correlates of perceived positive physical change, only greater increase in depressive symptoms (*B* = 0.70, 95% CI: 0.32, 1.09, *p*-value < 0.001, *R* ^2^ = 11%, β, 0.40) was a significant predictor of lower perceived positive physical change.

**Table 7:** Longitudinal associations between change in indicators of cognitive, mental, and physical health and physical change.

Change score	Model 1. Unadjusted linear regression			Model 2. Adjusted linear regression			Model 3. Multivariable linear regression including all predictors		
	*B* (95% CI), *p*-value	β	*R* ^2^	*B* (95% CI), *p*-value	β	*R* ^2^	*B* (95% CI), *p*-value	β	*R* ^2^
Global cognition	−1.05 (−2.06, −0.05), 0.39	−0.21	0.04	−1.06 (−2.04, −0.08), 0.035	−0.21	0.05	−0.43 (−1.46, 0.60), 0.413	−0.08	0.01
Memory complaints	−0.03 (−0.19, 0.13), 0.704	−0.03	0.001	−0.01 (−0.18, 0.15), 0.873	−0.01	0.001	−0.17 (−0.37, 0.03), 0.091	−0.17	0.03
Anxiety symptoms	0.39 (0.14, 0.65), 0.002	0.24	0.06	0.36 (0.11, 0.62), 0.006	0.22	0.05	0.04 (−0.29, 0.37), 0.821	0.02	0.001
Depressive symptoms	0.56 (0.31, 0.80), 0.001	0.34	0.12	0.55 (0.30, 0.79), 0.001	0.33	0.11	0.70 (0.32, 1.09), 0.001	0.40	0.11
Number of health conditions	0.20 (−0.12, 0.53), 0.214	0.10	0.01	0.14 (−0.19, 0.46), 0.403	0.06	0.01	−0.09 (−0.53, 0.34), 0.669	−0.04	0.002
Self-rated health	0.98 (−1.47, −0.48), 0.001	−0.29	0.08	−1.01 (−1.50, −0.52), 0.001	−0.30	0.09	−0.61 (−1.22, 0.02), 0.056	−0.19	0.03

Models 2 and 3 are adjusted for age, sex, marital status, and occupation before retirement. *N* = 174. *B* = Unstandardized regression coefficient. β = Standardized regression coefficient.

## Discussion and implications

This study tested cross-sectional associations between a range of objective and subjective indicators of cognitive, mental, and physical health (i.e., global cognition, memory complaints, anxiety symptoms, depressive symptoms, number of health conditions, and self-rated health) and scores on the AAQ’s subscales (i.e., assessing perceived psychological growth, psychosocial loss, and positive physical change) in a sample of very old Australians. The study also investigated whether 12-year change in the same health indicators was associated with scores on the AAQ’s subscales. Although numerous studies have linked health indicators to SPA, this is one of the few including, in the same model, subjective, objective, and informant-rated indicators of health as possible correlates of a multidimensional measure of SPA. In doing so, it allows us to identify those health indicators that are significant correlates of SPA subscales above and beyond the effect of other health indicators.

The first study hypothesis was partially confirmed as AAQ subscales were cross-sectionally associated with some but not all health indicators. Moreover, different AAQ subscales were associated with different health indicators. Starting from the AAQ subscale assessing psychological growth, among the investigated health indicators, the study found that more memory complaints and more depressive symptoms showed small cross-sectional associations with lower (worse) perceived psychological growth. When including all significant cross-sectional correlates in a multidimensional model, more depressive symptoms were the only cross-sectional independent variable explaining a significant yet small amount of cross-sectional variance (4%) in perceived psychological growth. Hence, concurrent depressive symptoms may be one key factor explaining the low experience of psychological growth in very old age. These results are aligned with a large amount of literature based on cross-sectional studies linking depressive symptoms to less positive SPA (e.g., 10). These results also support hypothesis 4, stating that the association between health indicators and the AAQ subscale is stronger when the domain of the health indicator (i.e., depression) and that of the AAQ subscale (i.e., psychological growth) match.

With regard to the AAQ subscale assessing perceived psychosocial loss, among the investigated health indicators, more anxiety symptoms and poorer self-rated health were associated at the cross-sectional level with greater (worse) perceptions of psychosocial loss. When including all significant correlates in a multidimensional model, only anxiety symptoms explained a significant amount of variance (9%) in psychosocial loss. These results are aligned with previous literature linking anxiety symptoms to more negative SPA (5, 10, 13). Again, these results are supportive of hypothesis 4.

Finally, with regard to perceived positive physical change, among the investigated health indicators, higher scores on global cognition, lower scores on anxiety symptoms, and better self-rated health were cross-sectionally associated with higher (better) scores for perceptions of physical change. In the multidimensional model, only higher self-rated health showed cross-sectional associations of moderate size with higher scores on perceived physical change. The finding that global cognition was no longer a significant independent variable of perceived physical change in the multidimensional model may be due to having included in the model indicators of physical health that are more strongly related to the dependent variable assessing perceived physical health. It may be that individuals are less aware of a small decline/change in their cognitive difficulties compared to being aware of a health change (e.g., diagnosis of a new health condition). Previous studies also found change in cognition as a less of an important contributor to SPA (2). The stronger association between the AAQ subscale assessing physical change and indicators of physical health compared to cognition may also be due to the matched content between assessments of physical health and perceived physical change (again, this result supports hypothesis 4) (Levy and Leifheit-Limson, 2009). However, previous studies that have assessed objective cognition and perceived age-related changes (SPA) in cognition also found stronger association between SPA and physical health indicators compared to cognition (4, 11). Hence, taken together, our cross-sectional results and previous evidence suggest that cognition is less of an important cross-sectional predictor of SPA and aspects of mental and physical health seem to be more important.

Better self-rated health has been previously related to more positive SPA (5, 10). Informant-rated disability is a more novel indicator found to be related to SPA. Interestingly, the number of diagnosed health conditions people had was not significantly related to SPA. This may be due to different health conditions impairing individuals to different degrees. In contrast, the questionnaire we used to assess disability covers several aspects of one’s daily functioning including physical health, functional abilities, cognitive abilities, and social and community engagement. Hence, this may better capture the impact that health conditions have on people’s life and, consequently, on how they perceive their own aging (hypothesis 3). The finding that number of physical health conditions—a more objective indicator of physical health—was not significantly associated with perceived physical change, whereas self-rated health—a subjective indicator of physical health—was associated with perceived physical change supports our third hypothesis and is aligned with previous cross-sectional and longitudinal evidence (25, 39). Overall, cross-sectional findings provided partial confirmation of hypothesis 1 and confirmation of hypotheses 3 and 4.

Moving to longitudinal results, contrary to our second hypothesis, 12-year change scores in none of the investigated health indicators were significantly associated with perceived psychological growth at follow-up. It is possible that psychological growth is related to variables we did not investigate in this study such as social relations, engagement with social activities, life experience, and life events. Correlates of psychological growth in very old age should be investigated in future studies as this is important to experience in older age.

In partial support of our second hypothesis, among the investigated health indicators, lower scores in 12-year change in anxiety symptoms and depressive symptoms, and self-rated health, and larger 12-year change in informant-rated disability were all associated with greater psychosocial loss. In the multiple regression analysis, only the 12-year decrease in anxiety symptoms and the decrease in depressive symptoms were significantly associated with less psychosocial loss. This is expected as there is a match between mental health changes and what is assessed in the psychosocial loss scale of the AAQ (in support of the fourth hypothesis). An increase in depressive symptoms over 20 years was also found to be associated with more negative SPA in previous longitudinal studies of very old German individuals (25). An increase in depressive and anxiety symptoms over 1 year was found to be related to more negative SPA in different age groups, including very old individuals living in the UK (11).

Also in partial support of our second hypothesis, among the investigated health indicators, 12-year decrease in global cognition, increase in anxiety symptoms, increase in informant-rated disability, and decrease in self-rated health were significantly associated with scores on the AAQ subscale positive physical change at follow-up (second hypothesis partially confirmed). In the multiple regression analysis, 12-year increase in depressive symptoms and that in informant-rated disability were significantly associated with lower scores on the AAQ subscale positive physical change. Hence, this study found a match between informant-rated disability and score on perceived physical change. A previous study found associations between greater functional difficulties over time and more negative SPA in very old individuals (27). Similar to the cross-sectional level, an increase in the number of diagnosed health conditions was not significantly related to SPA. This despite previous studies having found that newly diagnosed health conditions (e.g., cancer and cardiovascular events) predicted SPA (26). Again, it may be that the assessment of the impact that health conditions have on people’s life and functioning through a measure of disability better explains variability in levels of SPA, especially when all predictive variables are included in the same model.

In sum, also at the longitudinal level, we found that different subscales of the AAQ were associated with change scores in different health indicators. This highlights, once more, the importance of using multidimensional measures of SPA that provide separate scores for different domains of self-perceptions of age-related changes. In the case of our study, this made it possible for us to find that whereas changes in mood are relevant for perceived psychosocial losses, changes in informant-rated disability and depression are important for physical change. Moreover, other factors, rather than health indicators, may be important for perceived psychological growth. Overall cross-sectional and longitudinal study results suggest that individuals’ SPA may be related to their current mental and physical health and functioning, and they may be related to the changes they have been experiencing in their mental health and in their daily functioning over time.

Finally, although this was not a main objective of this study, we noticed that, in this study, people had some depressive symptoms in their 70s and experienced, on average, an increase in depressive symptoms over 12 years. Those who experienced a greater increase in depressive symptoms also had more negative perceptions of physical change at follow-up. It may be that depressive symptoms over time act as risk factors for both physical health and poorer SPA (48, 49). Hence, addressing depressive symptoms in old age may help prevent an increase in depressive symptoms in very old age, as well as prevent or lower decline in physical functioning and high levels of negative SPA. This hypothesis needs to be addressed in future studies.

The strengths of the study include (1) the 12-year length of the follow-up, (2) the focus on very old individuals, (3) the assessment of a range of health indicators comprising both objective and self-reported indicators and the inclusion of all of them in the same regression model, and (4) the use of a multidimensional measure of SPA. The limitations of the study include (1) assessment of AAQ only at follow-up, which did not make it possible for us to investigate whether change in health corresponds to change in AAQ; (2) an almost entirely Caucasian sample; and (3) selectivity of the study sample attrition between wave 1 and wave 7 of MAS. Thus, it is possible that only heathier participants, who are in theory more likely to have positive experiences with aging, reamained in the study sample at 12-year follow-up. Future research should continue to explore the complex interplay between various health indicators and SPA, with a focus on diverse populations and longitudinal changes in SPA.

## Conclusions

Overall study results suggest that individuals’ SPA may be related to their current mental and physical health, and they may be related to the changes they have been experiencing in their mental health over time. Cognition is a less important correlate of SPA, once having accounted for mental and physical health. Previous studies also found change in cognition as a less important correlate of SPA (2).

## Data Availability

The raw data supporting the conclusions of this article will be made available by the authors, without undue reservation.
