# Supplementary material for: Levels and changes in cognitive, mental, and physical health as correlates of attitudes to aging in very old age
Source: Front Psychiatry. 2025 Jul 11;16:1567754. doi: 10.3389/fpsyt.2025.1567754 (PMC12290458; doi:10.3389/fpsyt.2025.1567754)
Supplement: Supplementary file 1 [file DataSheet1.zip › Supplementary Table 6.DOCX]

| **Supplementary Table 6.**  *Cross-Sectional Associations Between Indicators of Cognitive, Mental, and Physical Health and Psychological Growth.* | | | | | | | | | |
| --- | --- | --- | --- | --- | --- | --- | --- | --- | --- |
| **Psychological Growth as Outcome** | | | | | | | | | |
|  | Model 1. Unadjusted linear regression | | | Model 2. Adjusted linear regression | | | Model 3. Multivariable linear regression including all predictors | | |
|  | B (95% CI), *p*-value | ß | R^2^ | B (95% CI), *p*-value | ß | R^2^ | B (95% CI), *p*-value | ß | R^2^ |
| Global cognition | 0.61 (-0.35, 1.56), .209 | 0.12 | 0.02 | 0.70 (-0.28, 1.67), 158 | 0.14 | 0.02 | 0.61 (-0.49; 1.70), .275 | 0.12 | 0.01 |
| Memory complaints | -0.004 (-0.01, -0.001), .003 | -0.23 | 0.05 | -0.004 (-0.01, -0.002), .002 | -0.24 | 0.06 | 0.10 (-0.17; 0.36), .461 | 0.08 | 0.01 |
| Anxiety symptoms | 0.02 (-0.28, 0.32), .903 | 0.01 | 0.001 | 0.03 (-0.28, 0.33), .872 | 0.01 | 0.002 | 0.21 (-0.25; 0.66), .366 | 0.10 | 0.01 |
| Depressive symptoms | -0.28 (-0.54, -0.02), .036 | -0.17 | 0.03 | -0.28 (-0.55, -0.02), .036 | -0.17 | 0.03 | 0.001 (-0.01; 0.01), .764 | 0.03 | 0.001 |
| Number of health conditions | 0.30 (-0.15, 0.74), .190 | 0.10 | 0.01 | 0.31 (-0.14, 0.77), .178 | 0.10 | 0.01 | 0.26 (-0.29; 0.81), .354 | 0.10 | 0.01 |
| Self-rated health | 0.49 (-0.19, 1.17), .156 | 0.11 | 0.01 | 0.47 (-0.22, 1.16), .177 | 0.11 | 0.01 | 0.64 (-0.40; 1.68), .224 | 0.14 | 0.02 |
| *Note.* Models 2 and 3 are adjusted for age, sex, marital status, and occupation before retirement. N= 174. B= Unstandardized regression coefficient. ß= Standardized regression coefficient. | | | | | | | | | |
