# Supplementary material for: Levels and changes in cognitive, mental, and physical health as correlates of attitudes to aging in very old age
Source: Front Psychiatry. 2025 Jul 11;16:1567754. doi: 10.3389/fpsyt.2025.1567754 (PMC12290458; doi:10.3389/fpsyt.2025.1567754)
Supplement: Supplementary file 1 [file DataSheet1.zip › Supplementary Table 5.docx]

| **Supplementary Table 5.**  *Correlations Among Study Variables.* | | | | | | | | | | | | |
| --- | --- | --- | --- | --- | --- | --- | --- | --- | --- | --- | --- | --- |
|  | 1. | 2. | 3. | 4. | 5. | 6. | 7. | 8. | 9. | 10. | 11. | 12. |
| 1. Perceived psychological growth | __ |  |  |  |  |  |  |  |  |  |  |  |
| 2. Perceived psychosocial loss | -0.01; .885 | __ |  |  |  |  |  |  |  |  |  |  |
| 3. Perceived physical change | 0.20; .009 | -0.17; .029 | __ |  |  |  |  |  |  |  |  |  |
| 4. Age | -0.05; .519 | 0.03; .736 | -0.15; 0.05 | __ |  |  |  |  |  |  |  |  |
| 5. Sex | -0.01; .856 | -0.16; .039 | -0.15; .048 | **-**0.01; .013 | **__** |  |  |  |  |  |  |  |
| 6. Marital status | 0.02; .835 | 0.08; .291 | -0.03; .718 | 0.12; .116 | 0.33; .001 | __ |  |  |  |  |  |  |
| 7. Occupation before retirement | 0.10; .197 | 0.07; .383 | -0.11; .165 | -0.08; .274 | 0.42; .001 | 0.20; .009 | __ |  |  |  |  |  |
| 8. Global cognition | 0.12; .209 | -0.11; 0.320 | 0.25; .012 | -0.31; .002 | 0.08; .418 | -0.01; .904 | 0.05; .598 | __ |  |  |  |  |
| 9. Memory complaints | -0.23; .003 | 0.10; .187 | -0.06; .411 | 0.04; .616 | -0.15; .042 | -0.002; .975 | -0.09; .237 | 0.01; .956 | __ |  |  |  |
| 10. Anxiety symptoms | 0.01; .903 | 0.28; .0004 | -0.26; 0.001 | 0.02; .777 | 0.21; .007 | 0.08; .290 | 0.26; .001 | -0.12; .241 | -0.13; .114 | __ |  |  |
| 11. Depressive symptoms | -0.20; .009 | -0.04; .6*43* | -0.06; .408 | 0.09; .259 | -0.02; .802 | 0.06; .449 | 0.09; .204 | -0.16; .109 | 0.26; .001 | 0.03; .699 | __ |  |
| 12. Number of health conditions | 0.07; .333 | 0.12; .128 | -0.06; .449 | 0.21; .005 | -0.12; .114 | -0.02; .815 | -0.07; 0.36 | -0.12; .237 | 0.04; .571 | 0.13; .112 | -0.13; .143 | __ |
| 13. Self-rated health | 0.14; .068 | -0.19; .015 | .44; .001 | -0.09; .249 | -0.15; .042 | 0.01; .884 | -0.14; .084 | 0.32; .001 | -0.19; .012 | -0.32; .001 | -0.44; .001 | -0.20; .009 |
