# Supplementary material for: Levels and changes in cognitive, mental, and physical health as correlates of attitudes to aging in very old age
Source: Front Psychiatry. 2025 Jul 11;16:1567754. doi: 10.3389/fpsyt.2025.1567754 (PMC12290458; doi:10.3389/fpsyt.2025.1567754)
Supplement: Supplementary file 1 [file DataSheet1.zip › Supplementary Table 4.DOCX]

| **Supplementary Table 4.**  *Neuropsychological tests used to calculate cognitive domains and global cognition scores.* | |
| --- | --- |
| **Cognitive Domain** | **Test** |
| Attention/Processing speed | Digit Symbol-Coding (44)  Trail Making Test (TMT) A (45) |
| Memory | Logical Memory Story A delayed recall (46)  Rey Auditory Verbal Learning Test (RAVLT) (45)  RAVLT total learning; sum of trials 1-5  RAVLT short-term delayed recall; trial 6  RAVLT long-term delayed recall; trial 7  Benton Visual Retention Test recognition (47) |
| Verbal Memory | As above, but not including the Benton Visual Retention Test. |
| Language | Boston Naming Test – 30 items (48)  Semantic Fluency (Animals) (45) |
| Visuo-spatial | Block Design (44) |
| Executive function | Controlled Oral Word Association Test (FAS) (45)  Trail Making Test (TMT) B (45) |
