# Supplementary material for: Levels and changes in cognitive, mental, and physical health as correlates of attitudes to aging in very old age
Source: Front Psychiatry. 2025 Jul 11;16:1567754. doi: 10.3389/fpsyt.2025.1567754 (PMC12290458; doi:10.3389/fpsyt.2025.1567754)
Supplement: Supplementary file 1 [file DataSheet1.zip › Supplementary Table 3.DOCX]

| **Supplementary Table 3.**  *Descriptive Statistics at Wave 7 for Participants Who Completed the AAQ and Those Who Did Not* | | | |
| --- | --- | --- | --- |
|  | **Participants who completed the AAQ** | **Participants who did not complete the AAQ** | **p-value** |
| n | 174 | 137 |  |
| **Variables** |  |  |  |
| Global cognition, M (SD) | 0.49 (0.82) | 0.84 (0.77) | .023 |
| Missing | 71 | 98 |  |
| Memory complaints, M (SD) | 24.06 (3.35) | 24.11 (3.50) | .925 |
| Missing | 10 | 72 |  |
| Anxiety symptoms, M (SD) | 2.31 (2.13) | 2 (2.04) | .377 |
| Missing | 14 | 91 |  |
| Depressive symptoms, M (SD) | 2.87 (2.37) | 3.54 (2.59) | .099 |
| Missing | 16 | 91 |  |
| Number of health conditions, M (SD) | 2.6 (1.6) | 2.55 (1.63) | .694 |
| Self-rated health, M (SD) | 3.19 (0.91) | 2.91 (1.09) | .044 |
| Poor, n (%) | 4 (2.4) | 5 (7.8) |  |
| Fair, n (%) | 36 (21.7) | 21 (32.8) |  |
| Good, n (%) | 57 (34.3) | 18 (28.1) |  |
| Very good, n (%) | 62 (37.3) | 15 (23.4) |  |
| Excellent, n (%) | 7 (4.2) | 5 (7.8) |  |
| Missing | 8 | 73 |  |
