# Supplementary material for: Levels and changes in cognitive, mental, and physical health as correlates of attitudes to aging in very old age
Source: Front Psychiatry. 2025 Jul 11;16:1567754. doi: 10.3389/fpsyt.2025.1567754 (PMC12290458; doi:10.3389/fpsyt.2025.1567754)
Supplement: Supplementary file 1 [file DataSheet1.zip › Supplementary Table 2.DOCX]

| **Supplementary Table 2.**  *Descriptive Statistics at Baseline for Participants Who Completed the AAQ and Those Who Did Not* | | | |
| --- | --- | --- | --- |
|  | **Participants who completed the AAQ** | **Participants who did not complete the AAQ** | **p-value** |
| n | 174 | 137 |  |
| **Variables** |  |  |  |
| Age, M (SD) | 75.36 (3.66) | 77.13 (4.37) | .0001 |
| Sex, n (%) |  |  |  |
| Women | 104 (59.8) | 93 (67.9) | .140 |
| Men | 70 (40.2) | 44 (32.1) |  |
| Marital status, n (%) |  |  |  |
| Never married | 16 (9.2) | 21 (15.3) | .013 |
| Married de facto | 88 (50.6) | 44 (32.1) |  |
| Separated | 1 (0.6) | 2 (1.5) |  |
| Divorced | 18 (10.3) | 13 (9.5) |  |
| Widowed | 51 (29.3) | 57 (41.6) |  |
| Main occupation when working, n (%) |  |  |  |
| Manager admin | 25 (14.8) | 17 (13.2) | .292 |
| Professional | 64 (37.9) | 36 (27.9) |  |
| Associate professional | 10 (5.9) | 4 (3.1) |  |
| Tradesperson | 5 (3.0) | 4 (3.1) |  |
| Advanced clerical service | 19 (11.2) | 14 (10.9) |  |
| Intermediate clerical sales service | 25 (14.8) | 29 (22.5) |  |
| Intermediate prod transport | 2 (1.2) | 1 (0.8) |  |
| Elementary clerical, sales service | 6 (3.6) | 11 (8.5) |  |
| Labourers and related | 1 (0.6) | 1 (0.8) |  |
| Home duties | 12 (7.1) | 9 (7.0) |  |
| Missing | 5 | 3 |  |
| Race, n (%) |  |  |  |
| Caucasian | 172 (100) | 134 (99.3) | .442 |
| Other | 0 (0) | 1 (0.7) |  |
| Missing | 2 | 2 |  |
| Global cognition, M (SD) | 0.40 (0.87) | 0.14 (1.04) | .016 |
| Missing |  |  |  |
| Memory complaints, M (SD) | 23.59 (2.63) | 23.81 (2.53) | .465 |
| Missing | 0 | 5 |  |
| Anxiety symptoms, M (SD) | 0.85 (1.62) | 1.08 (1.86) | .254 |
| Missing | 4 | 7 |  |
| Depressive symptoms, M (SD) | 1.61 (1.39) | 1.92 (1.62) | .070 |
| Missing | 0 | 0 |  |
| Number of health conditions, M (SD) | 1.31 (1.18) | 1.23 (1.19) | .571 |
| Self-rated health, M (SD) | 3.61 (0.76) | 3.49 (0.87) | .051 |
| Poor, n (%) | 1 (0.6) | 0 (0) |  |
| Fair, n (%) | 7 (4.0) | 18 (13.1) |  |
| Good, n (%) | 70 (40.2) | 50 (36.5) |  |
| Very good, n (%) | 76 (43.7) | 53 (38.7) |  |
| Excellent, n (%) | 20 (11.5) | 16 (11.7) |  |
| Missing | 0 | 0 |  |
