# Supplementary material for: Levels and changes in cognitive, mental, and physical health as correlates of attitudes to aging in very old age
Source: Front Psychiatry. 2025 Jul 11;16:1567754. doi: 10.3389/fpsyt.2025.1567754 (PMC12290458; doi:10.3389/fpsyt.2025.1567754)
Supplement: Supplementary file 1 [file DataSheet1.zip › Supplementary Table 1.DOCX]

| **Supplementary Table 1.**  *Descriptive Statistics at Baseline for the Those Who Took Part at MAS Wave 7 and Those Who Did Not* | | | |
| --- | --- | --- | --- |
|  | **Wave seven participants** | **MAS participants who did not take part at wave 7** | **p-value** |
| n | 311 | 742 |  |
| **Variables** |  |  |  |
| Age, M (SD) | 76.14 (4.08) | 79.27 (4.80) | <.001 |
| Missing | 0 | 12 |  |
| Sex, n (%) |  |  |  |
| Women | 197 (63.3) | 353 (48.4) | .001 |
| Men | 114 (36.7) | 377 (51.6) |  |
| Marital status, n (%) |  |  |  |
| Never married | 37 (11.9) | 95 (13.1) | .897 |
| Married de facto | 132 (42.4) | 298 (41.0) |  |
| Separated | 3 (1.0) | 11 (1.5) |  |
| Divorced | 31 (10.0) | 79 (10.9) |  |
| Widowed | 108 (34.7) | 244 (33.6) |  |
| Main occupation when working, n (%) |  |  |  |
| Manager admin | 42 (14.2) | 106 (15.3) | .267 |
| Professional | 100 (33.9) | 193 (27.9) |  |
| Associate professional | 14 (4.7) | 38 (5.5) |  |
| Tradesperson | 9 (3.1) | 49 (7.1) |  |
| Advanced clerical service | 33 (11.2) | 77 (11.1) |  |
| Intermediate clerical sales service | 54 (18.3) | 125 (18.1) |  |
| Intermediate prod transport | 3 (1.0) | 15 (2.2) |  |
| Elementary clerical, sales service | 17 (5.8) | 46 (6.7) |  |
| Labourers and related | 2 (0.7) | 5 (0.7) |  |
| Home duties | 21 (7.1) | 37 (5.4) |  |
| Missing | 16 | 51 |  |
| Race, n (%) |  |  |  |
| Caucasian | 306 (99.7) | 714 (98.3) | .130 |
| Other | 1 (0.3) | 12 (1.7) |  |
| Missing | 4 | 16 |  |
| Global cognition, M (SD) | 0.29 (0.96) | 0.37 (1.10) | <.001 |
| Missing | 1 | 20 |  |
| Memory complaints, M (SD) | 23.69 (2.59) | 23.97 (2.61) | .107 |
| Missing | 5 | 30 |  |
| Anxiety symptoms, M (SD) | 0.95 (1.73) | 1.19 (1.93) | .062 |
| Missing | 11 | 31 |  |
| Depressive symptoms, M (SD) | 1.75 (1.50) | 2.49 (2.12) | <.001 |
| Missing | 0 | 16 |  |
| Number of health conditions, M (SD) | 1.28 (1.18) | 1.31 (1.35) | .704 |
| Self-rated health, M (SD) | 3.55 (0.81) | 3.23 (0.93) | .001 |
| Poor, n (%) | 1 (0.3) | 16 (2.2) |  |
| Fair, n (%) | 25 (8.0) | 131 (18.0) |  |
| Good, n (%) | 120 (38.6) | 317 (43.6) |  |
| Very good, n (%) | 129 (41.5) | 195 (26.8) |  |
| Excellent, n (%) | 36 (11.6) | 68 (9.4) |  |
| Missing | 0 | 15 |  |
