# Supplementary material for: Levels and changes in cognitive, mental, and physical health as correlates of attitudes to aging in very old age
Source: Front Psychiatry. 2025 Jul 11;16:1567754. doi: 10.3389/fpsyt.2025.1567754 (PMC12290458; doi:10.3389/fpsyt.2025.1567754)
Supplement: Supplementary file 1 [file DataSheet1.zip › Supplementary Table 8.DOCX]

| **Supplementary Table 8.**  *Cross-Sectional Associations Between Indicators of Cognitive, Mental, and Physical Health, and Physical Change.* | | | | | | | | | |
| --- | --- | --- | --- | --- | --- | --- | --- | --- | --- |
| **Physical Change as Outcome** | | | | | | | | | |
|  | Model 1. Unadjusted linear regression | | | Model 2. Adjusted linear regression | | | Model 3. Multivariable linear regression including all predictors | | |
|  | B (95% CI), *p*-value | ß | R^2^ | B (95% CI), *p*-value | ß | R^2^ | B (95% CI), *p*-value | ß | R^2^ |
| Global cognition | 0.99 (0.22, 1.78), .012 | 0.25 | 0.05 | 0.81 (0.02, 1.61), .046 | 0.20 | 0.04 | 0.28 (-0.56; 1.12), .511 | 0.07 | 0.004 |
| Memory complaints | -0.001 (-0.003, 0.001), .411 | -0.06 | 0.004 | -0.001 (-0.004, 0.001), .225 | -0.09 | 0.01 | 0.15 (-0.06; 0.35), .154 | 0.14 | 0.02 |
| Anxiety symptoms | -0.44 (-0.70, -0.18), .001 | -0.26 | 0.07 | -0.40 (-0.66, -0.14), .003 | -0.24 | 0.05 | -0.20 (-0.57; 0.16), .268 | -0.11 | 0.01 |
| Depressive symptoms | -0.001 (-0.003, 0.001), .408 | -0.06 | 0.004 | -0.01 (-0.003, 0.002), .545 | -0.05 | 0.002 | 0.003 (-0.003; 0.01), .362 | 0.09 | 0.01 |
| Number of health conditions | -0.13 (-0.47, 0.21), .449 | -0.06 | 0.003 | -0.12 (-0.46, 0.22), .475 | -0.05 | 0.003 | 0.07 (-0.35; 0.49), .730 | 0.03 | 0.001 |
| Self-rated health | 1.76 (1.24, 2.30), <.001 | 0.46 | 0.21 | 1.70 (1.18, 2.23), <.001 | 0.44 | 0.19 | 1.59 (0.80; 2.38), .001 | 0.42 | 0.14 |
| *Note.* Models 2 and 3 are adjusted for age, sex, marital status, and occupation before retirement. N= 174. B= Unstandardized regression coefficient. ß= Standardized regression coefficient. | | | | | | | | | |
