# Supplementary material for: Levels and changes in cognitive, mental, and physical health as correlates of attitudes to aging in very old age
Source: Front Psychiatry. 2025 Jul 11;16:1567754. doi: 10.3389/fpsyt.2025.1567754 (PMC12290458; doi:10.3389/fpsyt.2025.1567754)
Supplement: Supplementary file 1 [file DataSheet1.zip › Supplementary Table 7.DOCX]

| **Supplementary Table 7.**  *Cross-Sectional Associations Between Indicators of Cognitive, Mental, and Physical Health and Psychosocial Loss.* | | | | | | | | | |
| --- | --- | --- | --- | --- | --- | --- | --- | --- | --- |
| **Psychosocial Loss as Outcome** | | | | | | | | | |
|  | Model 1. Unadjusted linear regression | | | Model 2. Adjusted linear regression | | | Model 3. Multivariable linear regression including all predictors | | |
|  | B (95% CI), *p*-value | ß | R^2^ | B (95% CI), *p*-value | ß | R^2^ | B (95% CI), *p*-value | ß | R^2^ |
| Global cognition | -0.47 (-1.40, 0.46), .316 | -0.10 | 0.01 | -0.21 (-1.16, 0.74), .664 | -0.04 | 0.02 | -0.39 (-1.38; 0.61), .441 | -0.08 | 0.01 |
| Memory complaints | 0.002 (-0.001, 0.004), .186 | 0.10 | 0.01 | 0.001 (-0.002, 0.004), .452 | 0.06 | 0.003 | 0.18 (-0.06; 0.42), .142 | 0.15 | 0.02 |
| Anxiety symptoms | 0.54 (0.24, 0.83), <.001 | 0.28 | 0.80 | 0.65 (0.36, 0.95), <.001 | 0.11 | 0.34 | 0.57 (0.14; 1.00), .010 | 0.28 | 0.06 |
| Depressive symptoms | -0.001 (-0.003, 0.002), .643 | -0.04 | 0.001 | -0.001 (-0.003, 0.002), .621 | -0.04 | 0.001 | -0.01 (-0.01; 0.002), .197 | -0.13 | 0.02 |
| Number of health conditions | 0.30 (-0.09, 0.69), .128 | 0.12 | 0.01 | 0.25 (-0.14, 0.65), .203 | 0.10 | 0.01 | 0.24 (-0.26; 0.74), .336 | 0.10 | 0.01 |
| Self-rated health | -0.90 (-1.58, -0.22), .010 | -0.20 | 0.04 | -0.97 (-1.64, -0.29), .005 | -0.22 | 0.05 | 0.13 (-0.80; 1.06), .787 | 0.03 | 0.001 |
| *Note.* Models 2 and 3 are adjusted for age, sex, marital status, and occupation before retirement. N= 174. B= Unstandardized regression coefficient. ß= Standardized regression coefficient. | | | | | | | | | |
